# 3DMorph Automatic Analysis of Microglial Morphology in Three Dimensions from *Ex Vivo* and *In Vivo* Imaging

**DOI:** 10.1523/ENEURO.0266-18.2018

**Published:** 2018-12-10

**Authors:** Elisa M. York, Jeffrey M. LeDue, Louis-Philippe Bernier, Brian A. MacVicar

**Affiliations:** Department of Psychiatry, Djavad Mowafaghian Centre for Brain Health, University of British Columbia, British Columbia V6T 1Z3, Canada

**Keywords:** 3D, automatic analysis, imaging, MATLAB, microglia, morphology

## Abstract

Microglia are dynamic immune cells of the central nervous system, and their morphology is commonly used as a readout of cellular function. However, current morphological analysis techniques rely on either tracing of cells or two-dimensional projection analysis, which are time-consuming, subject to bias, and may ignore important three-dimensional (3D) information. Therefore, we have created 3DMorph, a MATLAB-based script that analyzes microglial morphology from 3D data. The program initially requires input of threshold levels, cell size expectations, and preferred methods of skeletonization. This makes 3DMorph easily scalable and adaptable to different imaging parameters or cell types. After these settings are defined, the program is completely automatic and can batch process files without user input. Output data includes cell volume, territorial volume, branch length, number of endpoints and branch points, and average distance between cells. We show that 3DMorph is accurate compared to manual tracing, with significantly decreased user input time. Importantly, 3DMorph is capable of processing *in vivo* microglial morphology, as well as other 3D branching cell types, from mouse cranial windows or acute hippocampal slices. Therefore, we present a novel, user-friendly, scalable, and semiautomatic method of analyzing cell morphology in 3 dimensions. This method should improve the accuracy of cell measurements, remove user bias between conditions, increase reproducibility between experimenters and labs, and reduce user input time. We provide this open source code on GitHub so that it is free and accessible to all investigators.

## Visual Abstract

**Figure:**
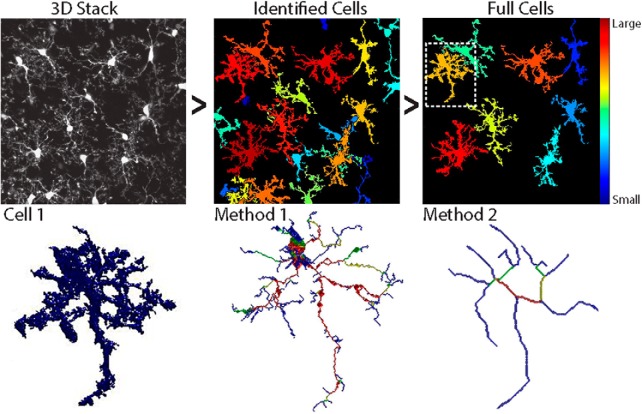

## Significance Statement

Microglial morphology is often considered to be an indicator of cellular activity, however current techniques to analyze morphology either lose valuable *z*-dimension information or are time intensive to perform. Therefore, we introduce 3DMorph, a MATLAB-based program that semi-automatically processes individual microglial morphology from overlapping 3D clusters, improving accuracy and processing time compared to current tools. 3DMorph is straightforward to use and adaptable to many imaging or experimental parameters. Once user settings are selected, 3DMorph can run in batch mode to automatically process multiple files. We validate 3DMorph against current techniques, and demonstrate the ability to detect differences in microglial morphologies from different *ex vivo* experimental conditions, as well as from *in vivo* data, and images of other branching cell types.

## Introduction

Microglia, the immune cells of the central nervous system, have small cell bodies and ramified processes that survey the local environment for signs of infection, damage, or disruption of molecular homeostasis (Nimmerjahn et al. 2005). In response to sensing damage, microglia rapidly extend their processes to converge at the site of injury (Davalos et al., 2005; Nimmerjahn et al., 2005; Hines et al. 2009; Drew et al., 2010; Dissing-Olesen et al., 2014; Eyo et al., 2014, 2015; Lou et al., 2016). On extensive damage of surrounding cells or stimulation by pathogen-associated triggers, microglia retract their processes to adopt an amoeboid morphology (Kreutzberg, 1996; Kloss et al. 2001; Doorn et al., 2014).

As a result of these contextual morphologic changes, microglial shape and process ramification have been used as correlates of cellular function (Davis et al. 1994; Karperien et al. 2013), with several methods developed to quantify their morphology. Current approaches include manually tracing processes throughout *z*-stack images (Baron et al. 2014; Takayama et al. 2016), or performing morphologic analysis on a two-dimensional (2D) maximum projection (Kozlowski and Weimer, 2012; Karperien et al., 2013; Torres-Platas et al., 2014; Verdonk et al., 2016). The first method is time-intensive and subject to experimenter bias and variability. The second technique loses three-dimensional (3D) information, leading to underestimation of process lengths or erroneous connection of processes. Three dimensional reconstructions of microglia cells can be generated using software such as Imaris (Perego et al. 2013; Erny et al., 2015); however, this is time-intensive and expensive. There is a clear need for a method that performs unbiased and automatic analysis of the 3D microglial structure observed in *ex vivo* and *in vivo* systems.

Here, we describe a method for semiautomatic analysis of microglial morphology in 3D using a custom MATLAB script, 3DMorph. The program uses graphical user interfaces to initially define image threshold, noise limits, and cell sizes. Once these settings are selected, a parameters file is saved that can be used to automatically batch process multiple files. From each image, an Excel file is saved with output data from the entire image (volume covered, average centroid distance), as well as from individual cells within the image (territorial volume, cell volume, cell ramification index, number of endpoints and branch points, and average, min, and max branch lengths).

The utility of 3DMorph is validated by analyzing and quantifying typical examples of morphologic changes of groups of microglia under control conditions, after hyper-ramification triggered by ATP application, and after retraction of ramifications triggered by inhibiting neuronal AMPA receptors with CNQX and action potentials with TTX. 3DMorph is also shown to process *in vivo* microglial images, as well as other branching cell types such as neurons. Therefore, this analysis software will allow for the automatic and unbiased analysis of microglial morphologies in 3D under several experimental and pathologic conditions.

## Materials and Methods

### Animal protocols

All housing and experimental procedures were conducted in accordance with University of British Columbia and Canadian Council on Animal Care regulations. CX3CR1^EGFP/EGFP^ or CX3CR1^+/EGFP^ mice on a C57Bl/6 background (Jung et al., 2000) were housed in a 12 h light/dark cycle with food and water *ad libitum*.

### Acute hippocampal slice preparation

Male mice (2 months of age) were anesthetized to surgical plane with isoflurane and decapitated according to protocols approved by the University of British Columbia committee on animal care. Brains were dissected and sliced horizontally with a vibratome (Leica VT1200S) at 300 μm thick in ice-cold *N*-methyl-d-glucamine (NMDG) slicing solution containing the following (in mm): 120 NMDG, 2.5 KCl, 25 NaHCO_3_, 1 CaCl_2_, 7 MgCl_2_, 1.2 NaH_2_PO_4_, 2 d-glucose, 2.4 sodium pyruvate, and 1.3 sodium l-ascorbate, which was constantly oxygenated with 95% O_2_ and 5% CO_2_. Hippocampal slices were immediately transferred to artificial CSF (aCSF) continuously oxygenated with 95% O_2_ and 5% CO_2_, and allowed to recover for 30 min at 32 ˚C. Artificial CSF contained the following (in mm): 126 NaCl, 2.5 KCl, 26 NaHCO_3_, 2 CaCl_2_, 2 MgCl_2_, 1.25 NaH_2_PO_4_, and 10 d-glucose, pH 7.3–7.4, osmolarity 300 mOsm.

### Treatment conditions and SNAPSHOT

Slices were incubated for 10 min at 32°C in either control aCSF, or aCSF containing 500 µm ATP, or 50 µm CNQX and 1 µm TTX. Slices were then fixed using the SNAPSHOT method (Dissing-Olesen and MacVicar, 2015), which consists of a 2 min immersion in 4% PFA at 80°C, rinse in 0.1 m PBS, and storage in clearing solution (0.1 m PBS with 20% DMSO, and 2% Triton X) at 4°C for 1 week. GFP fluorescence is well-preserved by this method, and slices were ready for imaging immediately after clearing.

### Acute hippocampal slice image acquisition

Fixed hippocampal slices were imaged with a two-photon Coherent Chameleon Ultra II laser with a Zeiss LSM 7 MP microscope. Using a Zeiss 20×-W/1.0 NA objective, GFP was excited at 920 nm, and emission was detected by a photomultiplier tube (Zeiss LSM BiG) after passing through a 535 ± 25 nm filter. Images were taken in the stratum radiatum of CA1 hippocampus at a depth of 150 µm ± 25 µm. Stacks were imaged at 16 bit, with 1024 × 1024 pixels, 16-line averaging, a zoom of 2.8, and *z*-step distance of 1 µm. After acquisition, background signal was removed from all images using Fiji’s rolling ball (radius = 25 pixels) background subtraction.

### Cranial window surgery

Mice were anesthetized using a tricomponent anesthesia (fentanyl, 0.05 mg/kg; midazolam, 5 mg/kg; medetomidine, 0.50 mg/kg), placed on a heating pad, and secured to a stereotactic frame. After the skull was exposed by removing the skin and periosteum, a circle was gently drilled into the skull’s surface at 0.5 mm lateral of −0.5 mm bregma. Once this portion of skull was removed, the brain was kept moist using surgical gel sponges in PBS (GelitaSpon). A custom-made 14-mm-diameter titanium ring was secured around the cranial window with light-curing dental cement (Heraeus). This ring fits into a custom-made head fixation plate, which secures the skull in the *x*, *y*, and *z* planes during *in vivo* imaging (Hefendehl et al., 2014).

### *In vivo* image acquisition

After cranial window preparation and titanium head ring fixation, anesthetized mice (fentanyl, 0.05 mg/kg; midazolam, 5 mg/kg; medetomidine, 0.50 mg/kg) were imaged on a custom-made two-photon microscope (Rosenegger et al., 2014) using a Coherent Chameleon Ultra II laser and a Zeiss 40×-W/1 NA objective. The head ring is secured to a fixation plate (Hefendehl et al., 2014), which is connected to a motorized *x*–*y* stage (Sutter Instruments). EGFP was imaged with 920 nm excitation and detected via non-descanned detectors after passing an ET525/50m-2P emission filter (Chroma Technology). Laser power did not exceed 45 mW throughout the experiment. *Z*-stack images (*z* = 40; 1 µm steps) were acquired at 512 × 512 pixels with no averaging, at a depth of 100–140 µm. Using a custom-designed perfusion system, aCSF was continuously perfused across the cortical surface at a rate of 3 ml/min. After acquisition, the signal of EGFP in these images was enhanced by increasing the contrast in Fiji, and motion artifacts were corrected with the Gaussian 3D filter.

### Neuronal dye loading

Layer 3 neurons from acute cortical slices (P24 rat) were whole-cell patch-clamped with borosilicate glass electrodes (3–4 MΩ). The intracellular recording solution consisted of the following (in mm): 113 K-gluconate, 2 MgCl_2_, 8 Na-gluconate, 3 KCl, 1 K_2_-EGTA, 4 K_2_-ATP, and 0.3 Na_3_-GTP at pH 7.25 with 10 HEPES. The solution also contained 50 µm AlexaFluor 594 hydrazide (ThermoFisher) to visualize the morphology of the dendritic arbor. The example cell was dialyzed with dye for 30 min before the patch electrode was slowly withdrawn before imaging. Images were post-processed in Fiji to subtract background using rolling ball radius, and enhance connectivity while removing speckles using the Gaussian Blur 3D filter and smooth functions.

### 3DMorph workflow

**Figure 1:**
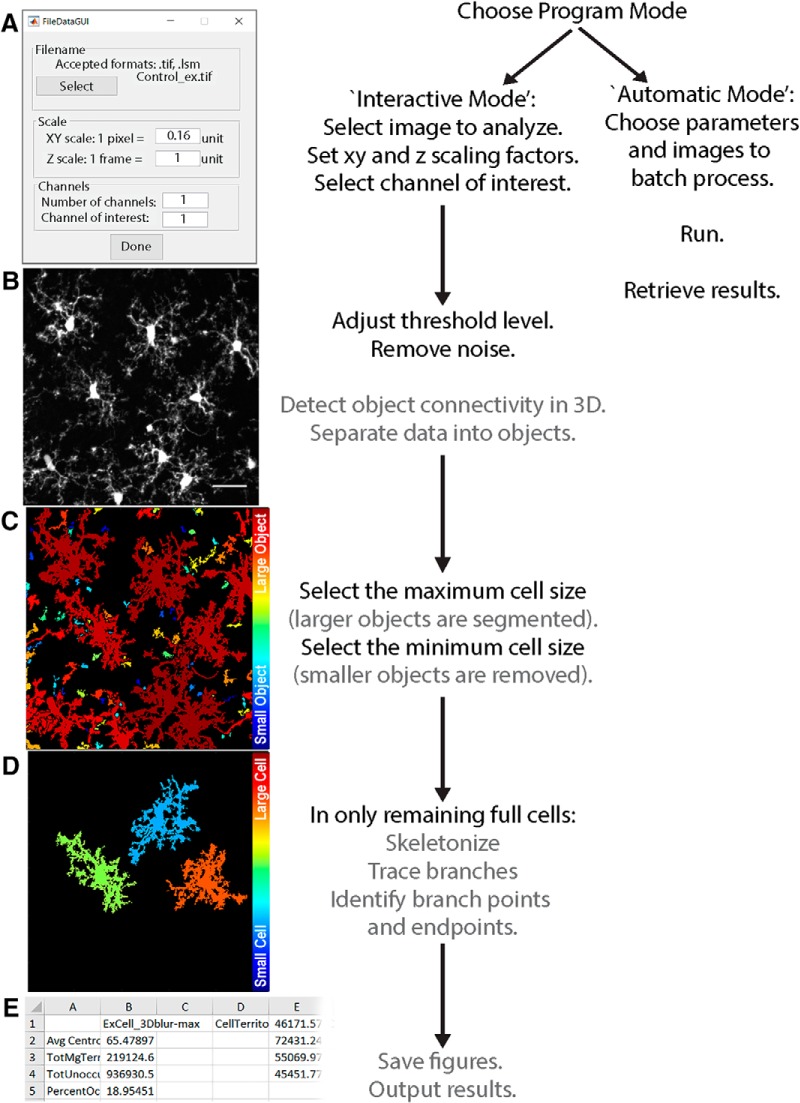
3DMorph workflow. The user selects either Interactive or Automatic mode. Interactive mode must be used first to generate a parameters file. ***A***, The user then selects the file to analyze, and specifies *x*, *y*, and *z* scale, number of channels, and the channel of interest. Both .tiff and .lsm files are supported. The original image (***B***) is loaded and 3D connected components (***C***) are automatically detected. ***D***, Large cells can be selected for segmentation and small objects can be removed. ***E***, After skeletonization and measurements of remaining cells, 3DMorph saves selected images and generates an Excel results file. Gray text indicates automatic steps.

The overall workflow of 3DMorph is outlined in Figure 1. Once images are acquired and processed as necessary, they should be moved to the Current Folder within MATLAB, or the data folder should be added to MATLAB’s search path. The user first selects “Interactive Mode” or “Automatic Mode”. Any time a new batch of images with different threshold settings or *x*-*y*-*z* scales are being processed, it is necessary to run the program in Interactive Mode. The user inputs the file of interest, its *x*, *y*, and *z* scale, the number of channels included in the image, and the channel of interest (Fig. 1A). Either .tiff or .lsm files are accepted. The user then adjusts threshold and size cutoff values (as discussed in detail below; Fig. 1*B*–*D*). Finally, a folder is created to store figures, and an Excel file of results is saved (Fig. 1*E*). Output values include data obtained before small object removal (average centroid distance between cells, total territorial volume, uncovered volume, percentage covered volume), and from individual full cells (cell and territorial volumes, ramification index, number of endpoints and branch points, and average, minimum, and maximum branch lengths). If the user selects the option, a separate Excel file containing a list of all branch lengths can be generated for each cell. Once an Interactive Mode analysis is complete, a parameter file will be created to save relevant input values. This can then be used to batch process a group of files using the same values and settings.

### Threshold images

**Figure 2:**
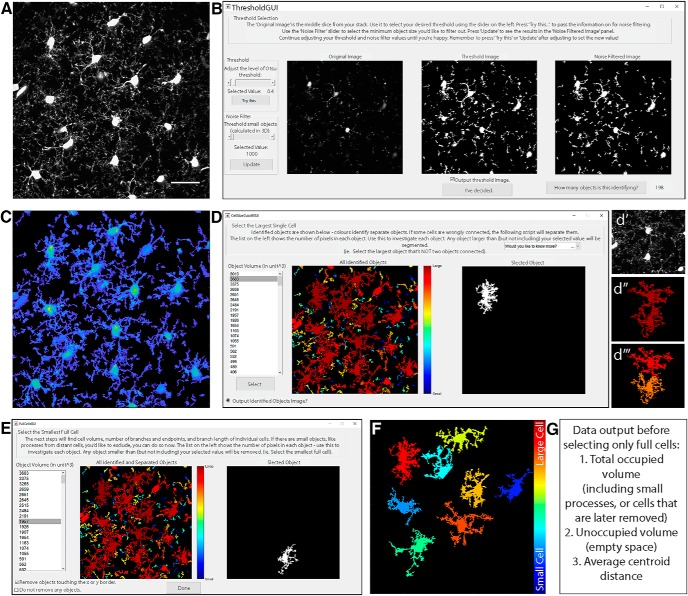
Select threshold and identify cells. ***A***, Grayscale maximum projection of original stack. Scale bar, 25 µm. ***B***, Select threshold level, and noise filter value to remove small spots. ***C***, “Output Threshold Image” is a 2D projection after threshold and noise filters are applied. To visualize 3D shapes of cells, hotter colors indicate thicker portions of cells. ***D***, 3DMorph automatically identifies 3D connected components, and the user selects a maximum cell size. Objects larger than this value are considered to be erroneously connected cells (*d*′, *d*‴), and will be segmented into separate objects (*d‴*). ***E***, Exclude remaining small cells, out-of-focus processes, or cells touching the *x*-*y* borders to isolate only full cells (***F***). ***G***, At this point, the program records total occupied volume (calculated before excluding small cells, processes, etc.), the unoccupied volume, and the distance between cell centroids.

Images saved as .lsm or .tiff files (Fig. 2*A*) are opened using the bfopen, or imread functions, respectively. A threshold value, based on Otsu method (Otsu, 1979) is then set. A new window will appear (Fig. 2*B*), showing the middle image of the *z*-stack, which can be used for a reference in deciding threshold values. A slider on the left sets the threshold level, and an automatically updated image shows the results of selected threshold values. The purpose of this step is to select a threshold level that accurately separates the small processes from background signal. Once an appropriate level is chosen, the “Try this…” button passes the threshold adjustment value to the noise filter. Again, a slider on the left can be adjusted to decide the minimum size of objects that should be considered noise. This filter functions in 3D, so if a process is removed here, it was likely separated from the cell in the thresholding step. Small cells and processes will be excluded in a later step (Fig. 2*E*), so it is not necessary to exclude them as noise here; they contribute to the calculation of total occupied brain volume. If selected, a threshold output image (Fig. 2*C*) can be generated, which is a projection of the thresholded *z*-stack, where the thicker portions of cells (such as somas) are displayed in yellow and thinner portions are in blue. This color coding is only used to visualize the approximate 3D shape of cells in a 2D image, and can be helpful to ensure the selected threshold value is correctly separating the cell from background signal.

### Identify and segment cells

The resulting thresholded image is separated into objects based on their 3D connectivity. To segment erroneously connected cells, identify the largest cell that is not two cells connected (Fig. 2*D*). Any object above this threshold (Fig. 2*d*′,*d*″) is automatically segmented by fitting a Gaussian mixture distribution (MATLAB function fitgmdist) to the data (Fig. 2*d*‴).

### Total territorial area of microglia

As microglia are highly ramified cells, the volume of brain they survey is greater than the volume of the cell itself. To estimate total territorial volume of microglia, a polygon is created to surround the outside points of each cell, and its volume is measured. All small cells and processes from above or below the image are included here. The amount of unoccupied volume and percentage of volume covered is also determined.

### Identify full cells

**Figure 3:**
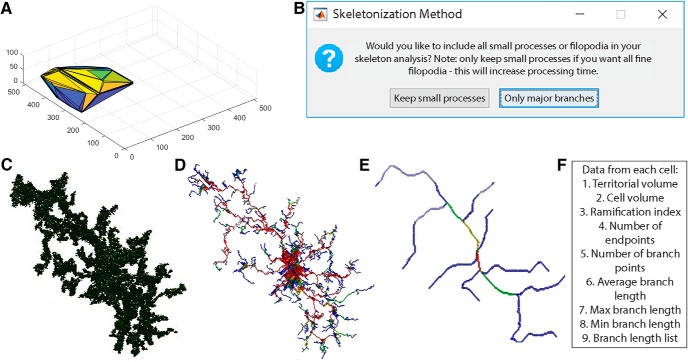
Analysis of individual full cells. ***A***, Territorial volume of each cell is determined by placing a polygon around all of the extreme points of the cell. ***B***, The user decides which skeletonization method to use and which images to save (including: original cell, skeleton, branch points, and end points). Each full cell (***C***) is then processed individually to generate a 3D skeleton, keeping either all branches (***D***), or only major branches (***E***). In skeleton figures, colors indicate order of connectivity (red = primary, yellow = secondary, green = tertiary, and blue = connected to endpoint). ***F***, After processing all cells, the program outputs territorial volume, cell volume, ramification index (calculated as territorial volume/cell volume), number of endpoints and branch points, as well as average, maximum, and minimum branch length for each cell. A complete list of branch lengths for each cell can also be generated.

To get accurate volume and branching data of individual microglia, it is important to eliminate cells that are partially excluded from the image. The user selects the smallest full cell (any smaller objects are removed from further processing), and indicates whether cells touching the *x*-*y* border should be removed (Fig. 2*E*). From the remaining cells (Fig. 2*F*), cell volumes and territorial volumes (Fig. 3*A*) are recorded. Cell volume is calculated by converting the number of voxels in each object to a real-world unit based on the specified scaling factors. Cell ramification index (or extent) is calculated by territorial volume divided by cell volume. This is a measure of how ramified or amoeboid the cells are. For instance, a small ramified cell and a bushy cell may have a similar cell volume, but the bushy cell will occupy more of its territorial space, therefore the ramification index measure will be smaller.

### Distance between cell centroids

Distribution of cells is addressed by measuring the average distance between centroids. Accurate centroids (unbiased by the “weight” of processes), are determined by eroding the cell to leave only the soma. These coordinates are converted to the unit of interest by multiplying the specified scales, and the distance between them is calculated. The average centroid distance is saved to the final results file.

### 3D skeletonization and branch tracing

To calculate branch lengths, endpoints, and branch points, a 3D skeleton of each cell is first generated. In 3DMorph, two skeletonization methods are available (Fig. 3*B*). The first keeps all small processes (Fig. 3*D*). This is ideal for images taken at a high magnification or to investigate differences in small filipodia-like structures. However, this method is also much slower and computationally demanding. This method is accomplished using the Skeleton3D method, developed by Kerschnitzki et al. (2013), and is available on File Exchange (https://www.mathworks.com/matlabcentral/fileexchange/43400-skeleton3d). Small extensions remaining on the skeleton, which are not likely to be true processes, are removed using the Graph2Skel3D and Skel2Graph3D (https://www.mathworks.com/matlabcentral/fileexchange/43527-skel2graph-3d).

The second skeleton method looks only at large branches and ignores smaller structures (Fig. 3*E*). This method might be preferred in images with a lower magnification and with several cells per image. It processes the skeleton using the Accurate Fast Marching method (available from the authors: https://www.mathworks.com/matlabcentral/fileexchange/24531-accurate-fast-marching).

Within each skeleton, endpoints are identified as pixels attached to only one other pixel. For each endpoint, a path between the endpoint and the centroid of the cell is traced to create a mask of each branch, from which the length is measured. This method may give a longer average branch length than other methods, as each end is traced to the soma, rather than to the nearest branch point. However, this method is more sensitive to differences in highly ramified versus bushy or amoeboid cells.

By adding the masks of all branches, a color code is generated with primary branches in red as those that have been traced four or more times, secondary branches in yellow have been traced three times, tertiary in green have been traced twice, and quaternary in blue have been traced only once (most distal process, which terminate in an endpoint). From this process mask, the number of branch points are calculated by determining points of intersection between primary, secondary, tertiary, or quaternary branches.

If requested, a new folder is generated in the Current Folder titled as “*filename*_figures” to save specified images and branch lengths. 3D representations of original cells (Fig. 3*C*), endpoints, branch points, and skeletons (Fig. 3*D*,*E*) can be saved. Images of initial thresholding, identified objects, segmented objects, and full cells will also be saved to this folder if the user chooses to have them generated.

### Export data

Finally, the data are written to an Excel file titled “Results*filename*” and saved to MATLAB’s Current Folder. For each image, the exported data includes: average centroid distances, total microglial territory volume, total unoccupied volume, and percentage of volume occupied. For each full cell: territory volume, cell volume, ramification index, number of endpoints and branch points, and average, minimum, and maximum branch lengths are saved.

### Statistics

All data were analyzed using a one-way ANOVA with a significance level of *p* < 0.05.

## Results

### Accuracy of 3DMorph results

**Figure 4:**
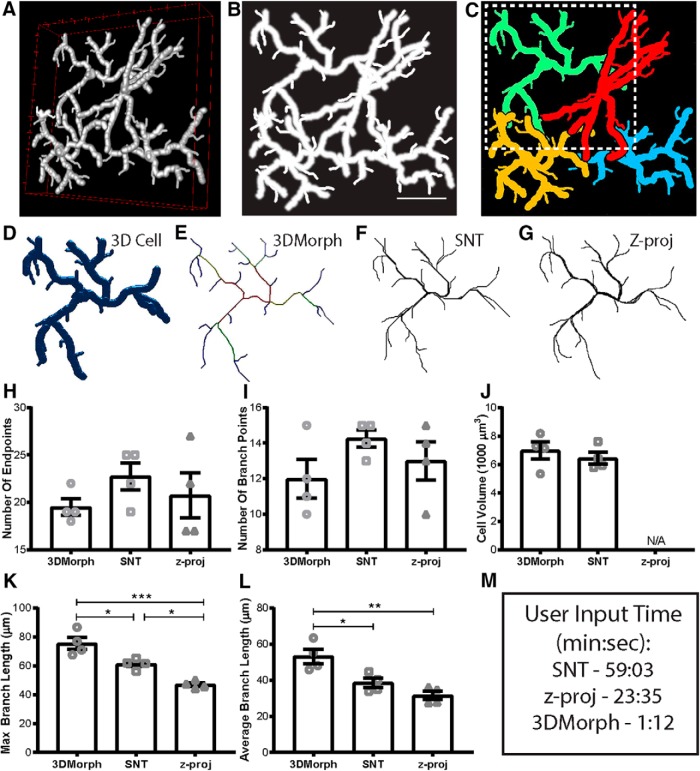
Validation and comparison of 3DMorph with current analysis tools. ***A***, 3D visualization of a manually-generated test image composed of four cells with overlapping processes. ***B***, *Z*-projection of test image. Scale bar, 25 µm. ***C***, Full cells as identified by 3DMorph. ***D***, A single full cell from the test image (outlined by dashed box in ***C***). ***E***, 3D skeleton generated automatically by 3DMorph. ***F***, 3D skeleton manually drawn using Simple Neurite Tracer (SNT). ***G***, 2D skeleton manually drawn using freehand tracing of a *z*-projection of the test image. Based on analysis by 3DMorph, SNT, or z-projection tracing, there is no significant difference in the number of endpoints (***H***) or branch points (***I***) recorded. ***J***, Cell volume measurements are accurate between 3DMorph and SNT, but unavailable from *z*-projection analysis. ***K***, Maximum branch length is significantly longer by 3DMorph and SNT analysis than by *z*-projection tracing. ***L***, Average branch lengths are significantly longer by 3DMorph than by SNT or *z*-projection. ***M***, Comparison of user input time to measure data. Error bars indicate mean ± SEM. *p < 0.05, **p < 0.01, ***p < 0.001 by one-way ANOVA.

To validate our 3DMorph program, we generated a test image to process and compare with current analysis methods (Fig. 4*A*,*B*). The image size is 512 × 512 pixels with 100 slices (0.21 µm/pixel, and 1 µm/slice). We analyzed this image in 3DMorph (Fig. 4*C*–*E*), as well as with the 3D-tracing ImageJ plugin, Simple Neurite Tracer (Fig. 4*F*), and by freehand tracing of a maximum intensity *z*-projection image (Fig. 4*G*). Features of each method are summarized in Table 1.

**Table 1.** 3DMorph, Simple neurite tracer ImageJ plugin, and freehand tracing of maximum z-projection images

	**3DMorph**	**Simple Neurite tracer**	***Z*-projection trace**
**Branch length**	✓	✓	✓
**Cell volume**	✓	✓	✘
**Territorial volume**	✓	×	✘
**Total occupied Volume**	✓	×	✘
**Ramification Index**	✓	×	✘
**No. of endpoints**	✓	✓	✓
**No. of branch points**	✓	✓	✓
**3D analysis**	✓	✓	×
**Automatic batch Processing**	✓	✘	×
**User input time**	Fastest (min)	Slowest (min–h)	Intermediate (10s of min)

Although both 3DMorph and simple neurite tracer process 3D information, only 3DMorph offers an automatic batch processing mode to greatly decrease user input time.

When comparing these techniques, there was no significant difference in the number of endpoints or branch points identified (Fig. 4*H*,*I*). However, process overlap in *z*-projected images led to greater uncertainty in separating branches of individual cells. Cell volumes were similar between 3DMorph and Simple Neurite Tracer (Fig. 4*J*), but are unavailable from *z*-projected data.

Maximum and average branch lengths were significantly greater when processed by 3DMorph compared to *z*-projected images (Fig. 4*K*,*L*), confirming the importance of maintaining 3D information. While Simple Neurite Tracer does analyze length in 3D, we found that these values are lower than our 3DMorph analysis. This is likely because 3DMorph measures the length of each endpoint to the soma centroid instead of the distance between an endpoint and its parent branch.

### Processing time of 3DMorph

In addition to providing similar or more accurate measurements, 3DMorph also took considerably less time for the same investigator to complete the analysis of the test image (1 min, 12 s) compared to Simple Neurite Tracer (59 min, 3 s) or *z*-projection tracing (23 min, 35 s; Fig. 4*M*). Although times were measured using Interactive Mode processing on one image, 3DMorph’s Automatic Mode processing would be even more advantageous.

Finally, although both Simple Neurite Tracer and *z*-projections require subjective branch tracing, 3DMorph completes these steps automatically. Therefore, variability between researchers will be greatly decreased, while improving data reproducibility among researchers and between labs.

### Microglial morphology changes in response to local cues

We next used 3DMorph to compare conditions which cause or mimic an increase or decrease in neuronal activity. As previously published (Dissing-Olesen et al., 2014), application of ATP triggers microglial process outgrowth, whereas processes retract in the presence of CNQX and TTX (Fontainhas et al., 2011). Although the biological pathways leading to these changes are interesting, here we do not address the biological cause of the process extensions or retractions. Instead we use these pharmacological manipulations only as tools to alter microglial morphology.

**Figure 5:**
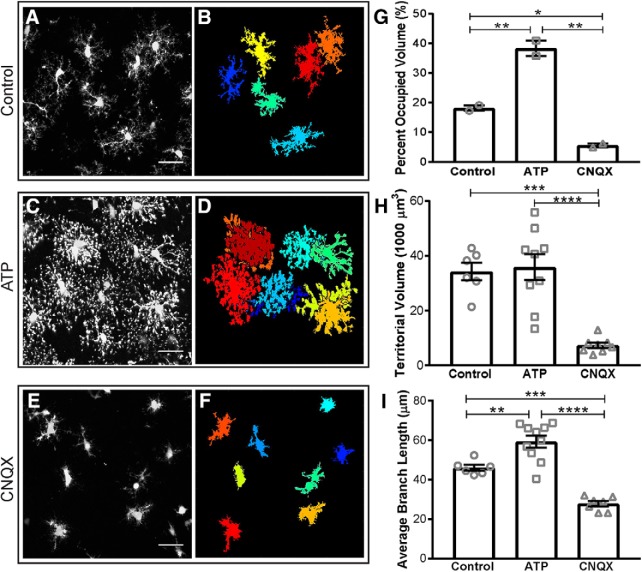
Microglia morphology changes in response to local cues. Microglia are incubated with control aCSF (***A***, ***B***), 500 µM ATP (***C***, ***D***), or 50 µM CNQX and 1 µM TTX (***E***, ***F***) before fixing and imaging (imaging dimensions: 174.08 × 174.08 × 50 µm). Original 3D projections (***A***, ***C***, ***E***; scale bar, 25 µm), and remaining full cells (***B***, ***D***, ***F***), are shown. ***G***, Quantification confirms that microglia cover more volume in ATP than in control conditions, while CNQX/TTX treatment decreases the total surveyed volume. When only full cells are considered, each microglial cell in CNQX/TTX conditions covers a smaller territorial volume (***H***) and has shorter average branch lengths (***I***) than control or ATP conditions, whereas ATP cells have significantly longer branch lengths than control. Error bars represent mean ± SEM. *p < 0.05, **p < 0.01, ***p < 0.001, ****p < 0.0001 by one-way ANOVA.

Acute hippocampal slices from CX3CR1^EGFP/+^ mice were incubated with either control aCSF (Fig. 5 *A*,*B*), 500 µM ATP (Fig. 5 *C*,*D*), or 50 µM CNQX with 1 µM TTX (Fig. 5 *E*,*F*) at 32°C. Slices were fixed using the SNAPSHOT protocol (Dissing-Olesen and MacVicar, 2015) and imaged by two-photon microscopy. A 1024 × 1024 image with 50 *z*-slices was acquired (*x*-*y* scale: 0.17 µm/pixel; *z* scale: 1 µm/slice; image dimensions, 174.08 × 174.08 × 50 µm). The same parameter file was used to automatically process images from these three conditions, removing any risk of experimenter bias.

3DMorph analysis revealed a significant increase in the percentage of brain volume surveyed by microglia in ATP conditions, whereas CNQX/TTX treatment decreased relative to control (Fig. 5*G*). When single cells were analyzed, the territorial volume of each cell treated with CNQX/TTX was significantly smaller than in control aCSF (Fig. 5*H*), whereas ATP induced a small increase. Finally, 3DMorph quantification confirmed that there is a significant increase in branch length of ATP-treated microglia, whereas CNQX/TTX-treated microglia have significantly shorter branches (Fig. 5*I*). These results demonstrate the ability of 3DMorph to automatically quantify morphologic changes of microglia *in situ* across different conditions in an automatic, unbiased, and reproducible manner.

### Microglial morphology *in vivo*

Although *in situ* imaging has many advantages, there is a growing push in the scientific field to confirm results using *in vivo* experiments. As 3DMorph requires only one channel containing the microglia image, and does not rely on counterstaining, it is possible to process microglia images from *in vivo* data.

**Figure 6:**
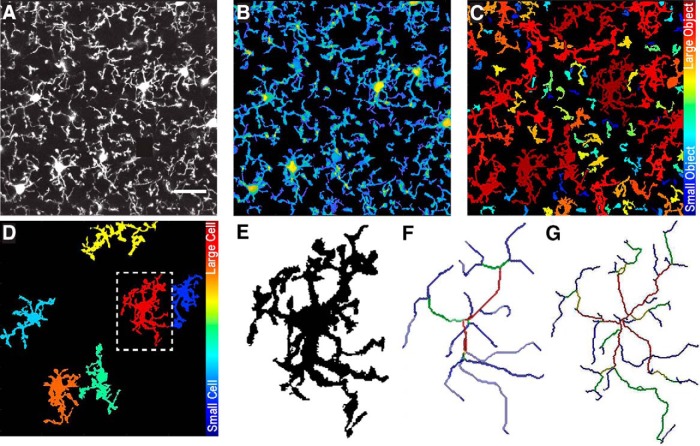
Morphology analysis of *in vivo* microglia images. ***A***, Maximum projection of *in vivo* image stack. ***B***, 3DMorph threshold image shown as a maximum projection. ***C***, Separation of thresholded image into individual objects, color-coded based on size of object. ***D***, Remaining full cells after removing small processes from out-of-frame cells. E, Isolated single cell from outlined region in ***D***. ***F***, Skeleton of major branches and (***G***) skeleton maintaining fine processes.

We confirm this by processing microglia images of CX3CR1^EGFP/EGFP^ mice acquired through a cranial window on an *in vivo* two-photon microscope (Fig. 6*A*). Image stacks were taken at 512 × 512 with 40 slices at an interval distance of 1 µm. 3DMorph analysis accurately thresholded (Fig. 6*B*), segmented (Fig. 6*C*), and skeletonized (Fig. 6*D*–*G*) these images, confirming that 3DMorph is appropriate for analyzing *in vivo* microglial morphology.

### Morphology of neurons

**Figure 7:**
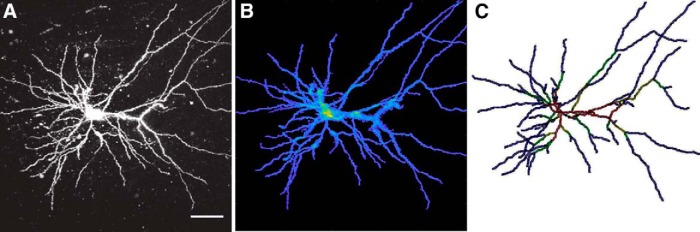
Morphology analysis of dye-loaded neuron. ***A***, Maximum projection of dye-loaded neuron. Scale bar, 50 µm. ***B***, Remaining cell following 3DMorph thresholding. ***C***, Skeletonized neuron keeping fine processes.

A benefit of 3DMorph’s Interactive Mode is that the software is adaptable to work with many types of input data. This makes it possible to process other types of branched cells in addition to microglia, such as neurons, astrocytes, or oligodendrocyte precursor cells. We validate 3DMorph’s performance in processing a patched and dye-loaded neuron (Fig. 7*A*). 3DMorph accurately identifies and maintains only the neuron (Fig. 7*B*), and skeletonizes the processes (Fig. 7*C*). Given the size difference between microglia and neurons, we therefore validate that 3DMorph correctly processes images of branched cells other than microglia.

## Discussion

Here, we present a novel method that allows for rapid and unbiased analysis of microglial morphologies in 3D. This is an open source script running in MATLAB, which is widely available through academic institutions, making 3DMorph free and easily accessible. We have written the program to make it user-friendly and compatible with many imaging settings. Furthermore, the program is well suited for analysis of other branched cells, such as astrocytes or oligodendrocyte precursor cells, in addition to microglia and neurons.

This program is an advancement to the currently available methods, as it relies on minimal user input, making it fast, replicable between experimenters and labs, and not subject to bias. Once parameters have been chosen, Automatic Mode processes large amounts of data with minimal input time. Importantly, using 3DMorph maintains the 3D information of cells, providing more accurate volume and branch length measurements.

We have validated 3DMorph against two other analysis techniques (Simple Neurite Tracer, and *z*-projection tracing), and detected alterations in microglial morphology, e.g., hyper-ramification triggered by ATP and hypo-ramification triggered by CNQX. Importantly, 3DMorph is compatible with images obtained *in vivo* and images of other branching cell types.

Another automatic 3D microglial analysis program has recently been published (Heindl et al., 2018). Although this is a powerful program offering a range of output results, our 3DMorph program offers some key advantages. Most importantly, it does not require a DAPI input image, which removes the necessity for immunostaining and makes 3DMorph capable of handling *in vivo* data. Furthermore, 3DMorph can process .tiff files, which allows the processing of images acquired by multiple types of software. We have also shown here that 3DMorph can manage thick sections of tissue and reliably separate cells that may appear overlapped when *z*-projected. Finally, although a completely automated analysis is available in 3DMorph, we first implement a series of graphical user interfaces that show real-time updates of how chosen settings will process the data. This transparency allows the user to confirm that the program is correctly processing their data.

Another promising analysis tool, ProMoIJ (Paris et al., 2018), has been recently published, which looks at microglial process motility. Although this method is excellent at analyzing tip extension and retraction, it does not analyze morphology differences of whole-cell images. Microglial morphology provides a clue to the cell’s biological function. Therefore, shape measurements (i.e., branch length and estimations of extent) are often used to differentiate healthy microglia from those associated with disease (Kreutzberg, 1996; Perego et al., 2013; Baron et al., 2014; Doorn et al., 2014; Torres-Platas et al., 2014). We encourage the field to use our 3DMorph program to perform their morphologic quantifications, and we gladly supply the original code (https://github.com/ElisaYork/3DMorph) so that it can be adapted and improved on by laboratories to best meet their needs.

### Troubleshooting

Although we have tried to make this program robust, user-friendly, and adaptable, it is possible issues may still occur. We have compiled this list of possible errors to assist with troubleshooting.

### Errors when running the script

1. Mex file error, or all output data for full cells is “0”:At the skeletonization step, if the script encounters an error, it will output zeros for this cell and move on to the next cell in the file. If all cells encountered an error, it is likely a mex compiling issue. Some skeletonization functions need to be compiled from C to MATLAB. In the original folder, go to: Functions>FastMarching_version3b>compile_c_files. You will need a compatible compiler to run this. If you do not have one, MATLAB will provide instructions on installing one.

2. ThresholdGUI:If you would like to keep the threshold and noise levels set to 0, please increase them, then move them back to 0. Be sure to confirm your adjusted values by pressing the “Try This” and “Update” buttons.

3. numObjMg = numel(FullMg):If you only have one cell in your image, you must choose to keep it during small cell removal by selecting the “Keep all cells” option. If this is not selected, your cell will be removed and pass on a blank image to the next processing step.

4. “waitbar” error:The program automatically generates waitbars to update you on how long it will take to process each step. It may reach an error if you have closed the waitbar window before it is finished processing.

5. GMModel: nuc must be a positive integer:During segmentation, the program erodes the connected cells to find nuclei and determine how many cells the object should be segmented into. If cells or nuclei are small (as in low magnifications), they may be eroded completely and a blank image will be passed on. In the first for loop of the Cell Segmentation portion of the script, decrease the value of se=strel(“diamond”,4); This will decrease the amount of erosion.

### Unsatisfactory data processing

1. 3DMorph works best on images with high signal-to-noise ratio. During the image acquisition stage, try using a high magnification of the cells you would like to analyze and decrease the *z*-slice interval so that branches remain connected in this dimension. If available, deconvolution post-processing may be helpful. It may also help to remove background before processing. This can be done with Fiji’s rolling ball radius subtraction.
2. If there is too much connectivity, the program will have trouble segmenting properly. Try increasing the threshold level so that fewer branches remain touching in the binary image.
3. If you observe too much connectivity that cannot be fixed by increasing threshold levels, try using a spatial sampling of 0.166 µm/pixel, for example a 1024 × 1024 pixel image with a size of 170 × 170 µm. Your input images can be scaled in ImageJ prior to analysis to match this pixel density. See ImageJ, Image menu scale function.
4. When batch processing images, it is beneficial to spend time finding parameters that work well for all files. Test a few example images in Interactive Mode to determine which settings are best. To save time, you can run the program until you have chosen a threshold, and large and small cell limit, then exit before it begins measuring skeletons. Once you have chosen suitable parameters, you will need to let the program run fully to generate a Parameters output file to use in your batch processing.
5. For accurate total image coverage, keep a low noise level so that small processes are still included. Small cells and processes from out-of-frame cells can be removed at a later step.

10.1523/ENEURO.0266-18.2018.ed1 Supplementary 1 Supplementary 3DMorph. Download Supplementary 1, TXT file

## Synthesis

Reviewing Editor: Maiken Nedergaard, University of Rochester Medical Center Decisions are customarily a result of the Reviewing Editor and the peer reviewers coming together and discussing their recommendations until a consensus is reached. When revisions are invited, a fact-based synthesis statement explaining their decision and outlining what is needed to prepare a revision will be listed below. The following reviewer(s) agreed to reveal their identity: Long-Jun Wu.

The authors provide a Matlabbased script that analyzes microglial morphology from 3D data called M3DMorph. The program requires certain input parameters defined after which it is possible to personalize its use. The advantage of is the analysis of cell morphology in 3 dimensions. The authors provide the open source code on GitHub and thereby accessible to all investigators.Reviewer #1 comments: This study described an analysis program named 3DMorph for automatic analysis of 3D morphology of microglial processes. The program is designed to analyze Z-stack microglial images from slice or living animals. It seems that the program has significant high-efficiency and low subjective bias compared with other analysis. Overall, the authors have invested considerable efforts to develop and validate the analysis program, which could be quite useful for studying microglial function in physiology and pathology. The major concern is that the study is a pure methodology paper with a quite narrow focus. 3D analysis of microglial morphology has also been pursued by several studies (Heindl et al, 2018; Paris et al., 2018), and thus the novelty of the study was compromised. I am surprised that the recent study on 3D analysis of microglia dynamics (Paris et al., 2018) was not discussed or even cited.
